# Extending Shelf-Life and Quality of Minimally Processed Golden Delicious Apples with Three Bioactive Coatings Combined with Cinnamon Essential Oil

**DOI:** 10.3390/foods10030597

**Published:** 2021-03-12

**Authors:** Gisselle Anahí Solís-Contreras, María Consuelo Rodríguez-Guillermo, María de la Luz Reyes-Vega, Cristobal N. Aguilar, Oscar Noé Rebolloso-Padilla, José Corona-Flores, Lluvia de Abril Alexandra Soriano-Melgar, Xochitl Ruelas-Chacon

**Affiliations:** 1 Department of Food Science and Technology, Autonomous Agrarian University Antonio Narro, Calzada Antonio Narro 1923, Saltillo 25315, Mexico; giss.soco23@gmail.com (G.A.S.-C.); consuelo_rodriguez95@hotmail.com (M.C.R.-G.); 2 Department of Research and Graduate Studies, Cerro de las Campanas, Autonomous University of Queretaro, Downtown Queretaro 76010, Mexico; luzrega@icloud.com; 3 Food Research Department, School of Chemistry, Coahuila Autonomous University, Saltillo 25280, Mexico; cristobal.aguilar@uadec.edu.mx; 4 Department of Animal Production, Autonomous Agrarian University Antonio Narro, Calzada Antonio Narro 1923, Saltillo 25315, Mexico; uaaan_lacteos@yahoo.com.mx; 5 Department of Planning, Autonomous Agrarian University Antonio Narro, Calzada Antonio Narro 1923, Saltillo 25315, Mexico; josedaniel.corona@gmail.com; 6 Department of Bioscience and Agrotechnology, CONACyT Research Fellow, Research Center for Applied Chemistry, Blvd. Enrique Reyna Hermosillo No. 140, Saltillo 25253, Mexico; alexandra.soriano@ciqa.edu.mx

**Keywords:** cinnamon essential oil, bioactive coatings, minimally processed apples, antioxidant agent

## Abstract

The application of coatings with essential oils for food preservation is an alternative way to keep minimally processed apple slices fresh, nutritious, safe, sensory palatable, and accessible for consumers. In the present study, the effect of three bioactive coatings on quality variables of minimally processed Golden Delicious apple slices for 25-days at 4 °C was evaluated. The coatings were CT1-chitosan-based, CT2-guar gum-based, and CT3-composite guar gum-starch-based; all three coatings contained cinnamon essential oil and were compared with UCT0-uncoated apple slices. The quality variables evaluated were weight-loss, firmness, browning index, total phenolic content, total soluble solids, titratable acidity, respiration rate, microbial analysis, and sensory evaluation. All coatings improved the preservation and sensorial quality variables of Golden Delicious apples; however, although the CT1-chitosan-based coating was capable of extending the shelf-life of minimally processed apple, it demonstrated less sensorially favorable scores for flavor, odor, and overall acceptance attributes.

## 1. Introduction

The lifestyle of today’s families and the concern for having a healthy, nutritious, and more natural diet has led to great commercial demand for minimally processed ready-to-eat-products such as fruits [1,2]. However, this type of food is readily perishable and difficult to preserve, given the processing to which they are subjected, which removes their natural coverings and causes sensory changes in the final product [3,4].

The use of new strategies and technologies to maintain and extend the shelf-life of these products is important in order to assure their availability in the market [5,6]. A strategy to reduce the deterioration of fresh-cut fruits, also known as minimally processed fruits, and to extend shelf-life is the use of edible coatings. Edible coatings are thin layers used on food matrices to reduce the loss of moisture and weight, tissue softening, respiration rate, multiple chemical reactions that generate microbiological spoilage, and quality deterioration due to their semipermeable barrier characteristics, as well as being useful carriers of food additives, e.g., antibrowning, antimicrobial, and antioxidant agents [3,4,7]. The efficacy of coatings depends mainly on the nature of their components. Another important characteristic is that they should be inexpensive, readily available, and should offer good mechanical and barrier properties. Various materials have proven successful as edible coatings, such as lipids, polysaccharides, and proteins, either alone or in combination [1,8,9].

Polysaccharides are commonly used to formulate coatings and films. Some of them include cellulose, chitosan, starch, carrageenan, zein, alginate, pectin, xanthan gum, and guar gum [6,10,11,12,13,14,15]. Chitosan (CS) is a polysaccharide obtained from the deacetylation of chitin, soluble in an acidic solution, with antimicrobial and antioxidant properties, and has several applications in food preservation and packaging [16]. Siripatrawan [17] and Kumar et al. [16] mention that the antioxidant activity, permeability, and mechanical properties of CS can be improved by the addition of natural antioxidants. Unfortunately, their use is limited because the addition of antioxidants, which compromises the odor and flavor of the food matrices. The application of CS to fruits and vegetables substantially extends their shelf-life [11].

Guar gum (GG) has also been used alone and combined with other polysaccharides for coatings and films for meat products, fruits, and vegetables [9,15,18,19]. GG is a polymer with high molecular weight, obtained from the plant *Cyamopsis tetragonoloba*. It is widely available and not expensive compared to other polymers; however, it provides poor mechanical properties and a high-water vapor permeability rate [18]. As an important approach towards reducing the inconveniences in the mechanical and permeability characteristics of the films and coatings, chemical treatments and the addition of several natural additives have been proposed [6,11,18,19]. GG forms homogenous films and emulsions that also entrap oil droplets in the aqueous medium by increasing viscosity [20].

Thakur et al. [6] mention that edible coatings and films are more efficient to improve the shelf-life and quality of fruits, vegetables, or food products when prepared in combination with other components as a composite base formulation as compared to their use as a single-based ingredient. Composite coatings have proven to be an economical alternative for food matrix uses. Some studies have reported that composite coatings developed with starch and polysaccharide gum have revealed successful results [21,22,23]. Starch (SCh) has been reported to be a good film-forming polysaccharide, as they are colorless, transparent, odorless, tasteless, and do not alter the natural appearance of the product, with low gas permeability, thus decreasing the respiration rate of pre-cut fruits and vegetables [1,15].

The polysaccharides mentioned above possess a hydrophilic character which makes the films and coatings acceptable barriers against gases (carbon dioxide and oxygen), but poor water vapor barriers [9,11]. Several lipids such as fatty acids, waxes, and essential oils (EOs) have been assessed as a way to increase the barrier properties of films and coatings. EOs from rosemary, oregano, citral, clove, cinnamon, garlic, orange thyme, and lemon are undeniably the most commonly applied, not only for their effects on barrier properties, but also as antioxidants and antimicrobials [9,11,18,19].

Cinnamon (*Cinnamomum zeylanicum*) is an antioxidant and antibrowning natural agent that is rich in essential oils [24]. Lu et al. [25] tested the antimicrobial activity of 45 essential oils on bacteria, fungi, and yeast, finding that the oil from cinnamon bark had a potent antimicrobial activity. The antimicrobial activity is attributed to cinnamaldehyde and eugenol. Kechichian et al. [26] and Souza et al. [24] reported that cinnamon essential oil showed strong antimicrobial activity against *Pseudomonas putida*, *Escherichia coli O157:H7*, fungi in bread, and pathogenic bacteria on several food products.

Little has been reported about the application of chitosan, guar gum, or composite coatings with as natural antibrowning and antimicrobial agents on minimally processed apple slices [6,11].

Currently, Golden Delicious apples represent 67% of the apples consumed in Mexico, and with an annual production of 47,769 metric tons for 2019/2020 [27]. Apple consumption in Mexico is driven by the retail sector, which represents 54% of domestic consumption. Consumers prefer Golden Delicious apples over other apple varieties for their sweetness, intense flavor, light crunch, color, and firmness [6,28]. Golden Delicious apples in the state of Coahuila, Mexico, represent 9% of the national production. Unfortunately, poor post-harvest handling, the use of pesticides, the presence of fungal pathogens, and the short shelf-life of this fruit represent an obstacle to expanding production, even with the use of refrigeration [29].

One of the main challenges facing the apple industry is maintaining post-harvest quality during storage, distribution, and sale to consumers so that the food is healthy and safe with natural additives. To respond and contribute to this challenge, the objective of this study was to evaluate the effect of three bioactive coatings combined with cinnamon essential oil as an antioxidant and antimicrobial agent on quality variables and the shelf-life of minimally processed Golden Delicious apple slices.

## 2. Materials and Methods

### 2.1. Materials

Golden Delicious apples at stage 5 of ripening of the ground color chart of the Post-Harvest Department of the University of California, Davis [30], were obtained from a commercial store in Saltillo, Mexico. Apples were visually sorted for uniformity in size, color, absence of fungal infection, and blemishes. Guar gum (GG), chitosan, corn starch, glycerol, citric acid, and tween-80 were obtained from Sigma-Aldrich Química, S. de RL. de CV., Mexico. Cinnamon essential oil was obtained from the Food Science and Technology Department from the Autonomous Agrarian University Antonio Narro.

### 2.2. Preparation of Minimally Processed Apples

A total of 324 apples were used for the experiment and of those 108 apples were processed per treatment. Fruits were washed with sodium hypochlorite solution (200 mg kg^−1^) for 2 min and air-dried at 22 °C ± 1 °C. Then, they were cut with a stainless-steel French fries’ cutter into equal slices of 1 cm^2^ × 7 cm in length. These pieces were subjected to blanching with hot water at 85 °C for 1.5 min. Subsequently, they were placed in an ice bath for 2 min and allowed to drain. After draining, an equal number of pieces were dipped for 2 min in coating solution (coated treatments) and water for control (uncoated treatment), all pieces were left to dry at room temperature for 30 min. Minimally processed apples were put in plastic trays (disposable polystyrene containers, Inix premium transparency, dimensions 7.48 in × 9.45 in × 1.57 in) and stored in controlled chambers at 4 °C and 85% relative humidity (RH). To simplify the study, the slices of each whole apple were used for the analyses of quality variables and each coating treatment during the 25-days storage period.

### 2.3. Bioactive Coating Formulations

Three coating formulations were prepared for the study (CT1, CT2, and CT3), and UCT0 (uncoated group, only dipped in distilled water). The CT1 formulation consisted of dissolving 1.5% of citric acid (*w/v*) in water with constant stirring for 5 min, adding 1.0% of chitosan (*w*/*v*), and constantly stirring for an hour at 50 °C and 0.02% tween-80 (*w*/*v*). Subsequently, 0.06% cinnamon essential oil (*v*/*v*) was added under continuous stirring until the emulsion was obtained. It was allowed to cool before applying it to the minimally processed apples. CT2 was prepared by dissolving 35% of glycerol (*v*/*w*) under constant stirring, then 10% cinnamon oil (*v*/*v*) was added, and stirring continued at 60 °C for about 15 min until the emulsion was formed. The temperature was lowered to 40 °C to be able to add 1.5% guar gum (*w*/*v*); stirring continued until an emulsion was formed. CT3 was a combination (50:50) of two formulations, CT2 and a formulation prepared by dissolving 10% cinnamon oil in water at 60 °C, then adding 1.0% corn starch and increasing the temperature to 80 °C, and maintaining this temperature for 10 min. All formulations were stirred at 800 rpm at the corresponding temperature on a magnetic stirrer/hot plate (Lab companion, HP-30100, Cole-Parmer, IL, USA). The composition of the treatments studied were: CT1, contained chitosan, citric acid, tween-80, and cinnamon essential oil; CT2, contained guar gum, glycerol, and cinnamon essential oil; and CT3, contained guar gum, glycerol, corn starch, and cinnamon essential oil.

### 2.4. Weight Loss Percentage

The apple slices samples subjected to UCT0, CT1, CT2, and CT3 were weighed in triplicate every 5-days during the 25-days storage period. The differences between initial and final slice weight gave the total weight loss during storage intervals and were expressed as percentages of the fresh weight [31].

### 2.5. Firmness Analyses

The firmness of the apple slices for each treatment was measured using a digital force gauge penetrometer (PCE-PTR 200, PCE group, Albacete, Castilla la Mancha, Spain) with a 6-mm plunger tip, with three readings per treatment at the equator of the slices. Results were expressed in Newtons (N) [31].

### 2.6. Assay of Color (Brown Index, BI)

Color was analyzed using a Minolta CR-400 colorimeter. The a*, b*, and L* values were used to obtain the browning index (BI) according to Borges et al. [32], Equation (1):

(1) $$\mathrm{BI}=\;\frac{100\cdot \left(X\;-0.31\right)}{0.172}\;\;X=\;\frac{\left(a\;+1.75L\right)}{5.64L+a\;-3.02b}$$

where three slices of apple were used for the color test, with six readings per analysis (two readings per slice) every five days during the storage period.

### 2.7. Total Phenolic Content

Following the Folin–Ciocalteu method described by Rocha and Morais (2002) [33] with slight modifications, the total phenolic content of the uncoated and coated apple slices was evaluated. By homogenizing 10 g of each sample in 80 mL of water and centrifugating at 3000 rpm for 20 min, 0.5 mL of the supernatant was mixed with 2.5 mL of Folin and Ciocalteu’s reagent, and 2 mL of sodium bicarbonate solution was added. Then, the samples were incubated first at 30 °C for one hour and then for another hour at 4 °C. Absorbance was measured at 760 nm with a spectrophotometer (Genesys 10 UV, Thermo Electron, Madison, WI, USA). Total phenols were expressed as mg of gallic acid equivalent (mgGAE)/g of fresh fruit for each treatment.

### 2.8. Total Acidity (TA) and Total Soluble Solids (TSS)

The TA was measured following the AOAC method [34], 10 g of apple of each treatment in triplicate were homogenized with 90 mL of distilled water, and then filtered. Twenty milliliters of each filtrate and 2 drops of phenolphthalein were titrated with 0.1 N NaOH and expressed as percentage of total acid. The TSS of the homogenized samples was measured with a digital refractometer (Atago, Tokyo, Japan) in triplicate and was expressed as °Brix [33].

### 2.9. Respiration Rate

The respiration rate of apple slices in each treatment was evaluated every five days during the 25-days storage period. Six slices of each treatment were randomly distributed in an airtight glass container with a capacity of 1.80 L under refrigerated conditions. Gas samples were taken from the container with a needle inserted through a septum fixed at the center of the jar lid; the needle was connected to a PDI Dansensor Gas Analyzer (Checkmate II, Ringsted, Denmark) as described by Ruelas et al. [31].

### 2.10. Microbial Analysis

The microbial analysis was conducted during the storage period of 25-days. Ten grams of samples of each treatment were homogenized for 1 min with casein peptone (90 mL) using a stainless-steel food processor (Nutribullet series 600-watt NBR-0804R, Nutribullet, LLC, Los Angeles, CA, USA). Serial dilutions were made in triplicate. The mesophilic bacteria counts were determined in plate count agar (PCA), and yeast and molds in potato dextrose agar (PDA), and visible colonies were counted as log CFU/g, following the procedure described by Song et al. [35].

### 2.11. Sensory Evaluation

In the sensory evaluation, the characteristics of overall appearance, color, odor, flavor, texture, and overall acceptance were evaluated. This test was conducted by a trained sensory panel of 30 judges from the Autonomous Agrarian University Antonio Narro, Saltillo, Mexico. The apple slices samples were presented on biodegradable plates (No. 855, dimensions 8.46 in × 5.98 in × 0.59 in, Fragoso group, Mexico) under normal lighting conditions in the cubicles at the sensory lab. The panelists evaluated the quality of the minimally processed apple slices with the application of the three treatments and the control, using the hedonic test with a seven-point scale (1 = strongly disliked, 2 = moderately disliked, 3 = slightly disliked, 4 = neither like nor disliked, 5 = slightly liked, 6 = moderately liked, 7 = strongly liked).

### 2.12. Statistical Analysis

The experiment was conducted according to a completely randomized design with a factorial arrangement. Data were analyzed by two-way analysis of variance (ANOVA) with three replications with storage time and treatments as factors, using Infostat version 2018 (Infostat.com.ar, Univ. Nac. De Cordova, Argentina), including a least significant difference test. The Fisher test was used to compare the mean values in different storage intervals. Statistical differences were declared at *p* < 0.05.

## 3. Results and Discussion

### 3.1. Weight Loss

**Figure 1:**
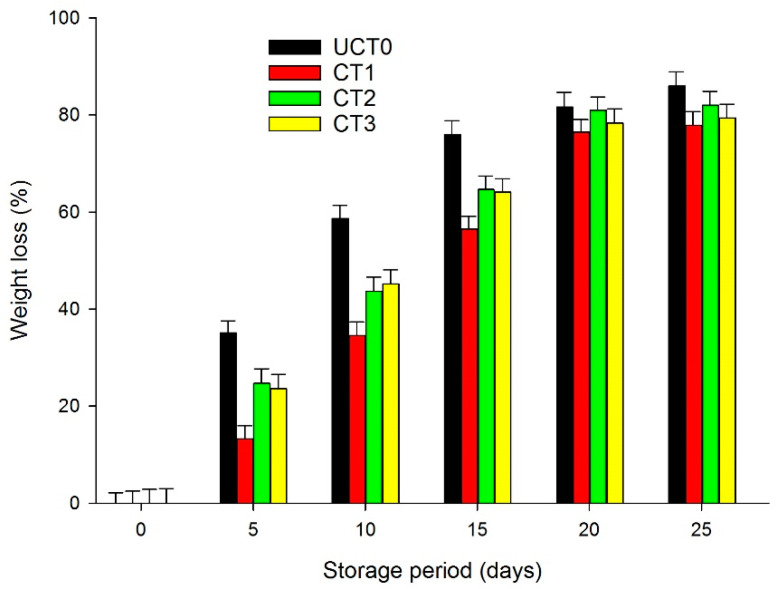
Weight loss (%) of Golden Delicious apple slices coated (with CT1, CT2, and CT3), and not treated (UCT0) at day 0, and after 5, 10, 15, 20, and 25-days of storage at 4 °C in dark conditions and 85% relative humidity (RH). Values are means ± standard deviations (*n* = 3). Abbreviations: UCT0, uncoated apple slices used as control; CT1, apple slices coated with chitosan-glycerol-cinnamon oil; CT2, apple slices coated with guar gum-glycerol-cinnamon oil; CT3, apple slices coated with composite guar gum-starch-glycerol-cinnamon oil.

Subjecting apples to the slicing process exposed the skinless tissue to an environment with lower relative humidity, which resulted in a considerable weight loss. Figure 1, shows the evolution of slice decay, in percentage, considering the initial mass during the 25-days storage period at 4 °C. Due to water vapor permeability, weight loss took place right after the first 5-days of storage. For CT1, CT2, and CT3, the barrier effect was evident, since the weight loss was lower in the CT2 and CT3 treatments, and much less in CT1, compared to UCT0. Both CT2 and CT3 reduced the rate of weight loss; however, there was a difference (*p* > 0.05) with CT1 because it reduced the weight loss of the sliced apples to a greater extent. Similar findings were reported by Cofelice et al. [8] with alginate/essential oil nanoformulations applied on fresh-cut apples during 14-days storage under cold conditions. Qi et al. [11] compared uncoated apple slices with chitosan-ascorbic acid-CaCl_2_-coated slices and chitosan-citric acid-CaCl_2_-coated slices, and after 2-days of storage, uncoated apple slices lost around 19% of their weight, while coated apple slices lost 15% of their weight, and the weight loss gradually continued until the end of the 8-days.

### 3.2. Firmness Analysis

**Figure 2:**
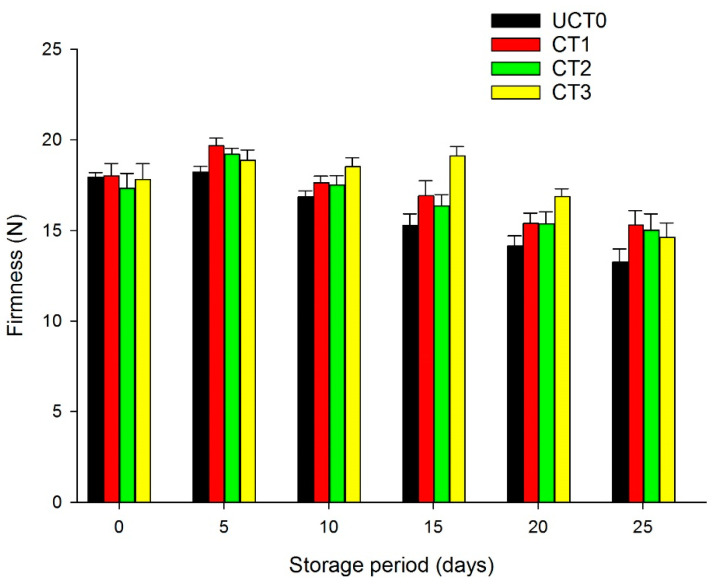
Firmness (N) of Golden Delicious apple slices coated (with CT1, CT2, and CT3), and not treated (UCT0) at day 0, and after 5, 10, 15, 20, and 25-days of storage at 4 °C in dark conditions and 85% relative humidity (RH). Values indicate the means ± standard deviations (*n* = 3). Abbreviations: UCT0, uncoated apple slices used as control; CT1, apple slices coated with chitosan-glycerol-cinnamon oil; CT2, apple slices coated with guar gum-glycerol-cinnamon oil; CT3, apple slices coated with composite guar gum-starch-glycerol-cinnamon oil.

An important quality variable for minimally processed apples is firmness; slicing them causes tissue softening. The loss of firmness is an important effect due to the action of hydrolytic and pectolytic enzymes on the pectic substances of the cell wall, the decreased crystallinity of cellulose, and thinning of the cell walls [11,36]. Data of the firmness analysis are shown in Figure 2. All treatments presented similar firmness on 5-days of storage (*p* > 0.05). On 10-days of storage, a significant deterioration in firmness was observed in UCT0 compared to the coated samples; CT1 and CT2 had similar values (*p* > 0.05), and CT3 had the greatest value for firmness. This demonstrated that the chitosan-based with cinnamon essential oil coating avoided tissue softening more efficiently compared with the other two coatings (CT1 and CT2), and this effect was maintained up to the 20-days of the storage period at 4 °C. Similar findings have been reported by Qi et al. [11], who mentioned that apple slices treated with chitosan-ascorbic acid-CaCl_2_ coating had the highest firmness. Sarengaowa et al. [36] studied the effect of different essential oils with an alginate-based coating with citric and ascorbic acid and found that firmness decreased during the storage for 16-days at 4 °C, and as expected there were significantly higher values for firmness on the coated sample than on the control.

### 3.3. Color, Brown Index (BI)

**Figure 3:**
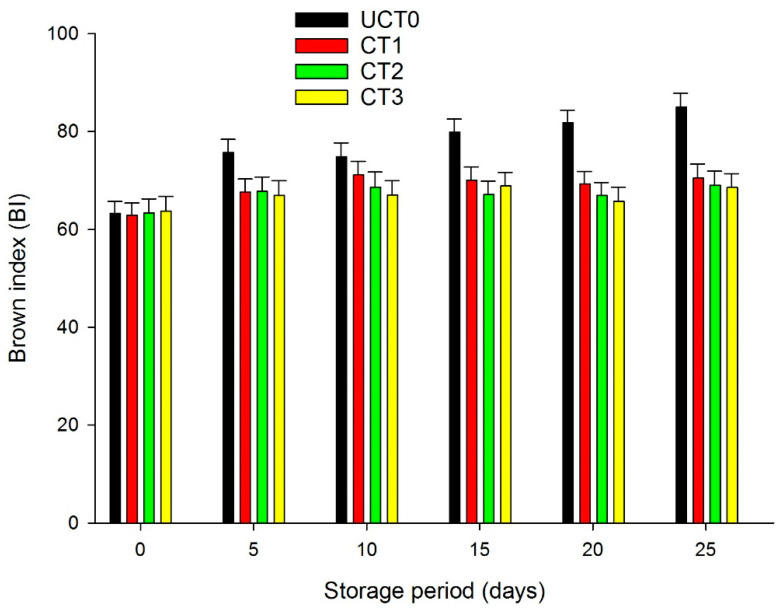
The brown index (BI) of Golden Delicious apple slices coated (with CT1, CT2, and CT3), and not treated (UCT0) at day 0, and after 5, 10, 15, 20, and 25-days of storage at 4 °C in dark conditions and 85% relative humidity (RH). Values indicate the means ± standard deviations (*n* = 3). Abbreviations: UCT0, uncoated apple slices used as control; CT1, apple slices coated with chitosan-glycerol-cinnamon oil; CT2, apple slices coated with guar gum-glycerol-cinnamon oil; CT3, apple slices coated with composite guar gum-starch-glycerol-cinnamon oil.

When peeled and cut, apples suffer damage to tissue cells, releasing polyphenol oxidases (PPO), which then bind with substrates and cause browning. Figure 3 illustrates that the uncoated slices presented higher browning index values during the storage period than the coated ones from day 5 of storage. Development of browning in apple slices gradually increased in UCT0, whereas in the coated slices a delay in the browning index was observed (*p* < 0.05). CT1, CT2, and CT3 showed similar BI values (*p* > 0.05) throughout the 25-days study. This effect explains that the CT1 coating for itself has antioxidant activity as well as the cinnamon oil, and CT2 and CT3 coatings possess cinnamon essential oil as a natural antioxidant agent. Alginate-CaCl_2_ and acetylate monoglyceride coating and alginate-CaCl_2_-butter solution coating reduced BI compared with the control apple slices [37]. Perez-Gago et al. [38], using composite edible coatings prepared with whey protein isolate (WPI), whey protein concentrate (WPC), or hydroxypropylmethylcellulose (HPMC) all three mixed with beeswax (BW) or carnauba wax (CarW) as coatings for apple slices during a 7-days trail and at 5 °C, demonstrated that the use of these coating formulations significantly reduced BI compared to the uncoated apples dipped in citric acid and sodium chloride solutions.

### 3.4. Total Phenolic Content

**Figure 4:**
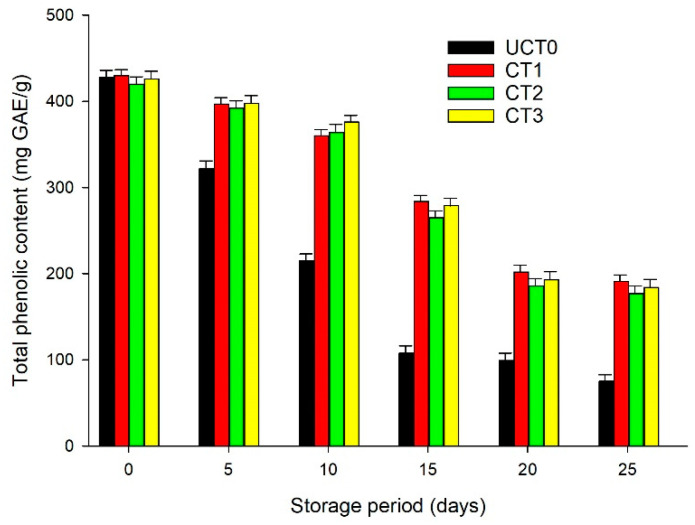
Behavior of total phenolic content (mg GAE/g) of Golden Delicious apple slices coated (with CT1, CT2, and CT3), and not treated (UCT0) at day 0, and after 5, 10, 15, 20, and 25-days of storage at 4 °C in dark conditions and 85% relative humidity (RH). Values indicate the means ± standard deviations (*n* = 3). Abbreviations: UCT0, uncoated apple slices used as control; CT1, apple slices coated with chitosan-glycerol-cinnamon oil; CT2, apple slices coated with guar gum-glycerol-cinnamon oil; CT3, apple slices coated with composite guar gum-starch-glycerol-cinnamon oil.

Apples are a good source of phenolic components with biological activities. The principal phenolic components of apples are chlorogenic acid, p-coumarin, caffeic acid, catechin, phlorizin, quercetin, rutin, and epicatechin, and the concentration of these phenolic components is extremely variable [39]. The minimal process operations like cutting and peeling of apples activate the phenylalanine ammonia lyases that stimulate the synthesis of phenols contrarily a decrease in levels of phenolic components is attributed to PPO [8]. Figure 4 illustrates the content of phenolic components in the apple slices. The loss of the total phenolic content in UCT0 was very notable during the 25-days trial (*p* < 0.05) when compared with any of the coated treatments. CT1 and CT3 maintained a similar behavior (*p* > 0.05) during the storage period related to the total phenolic content (TPC); however, in CT2 the TPC was lower than in CT1 and CT3 (*p* < 0.05). Thus, applying bioactive coatings considerably reduces the loss of total phenolic content.

### 3.5. Total Soluble Solids (TSS)

**Figure 5:**
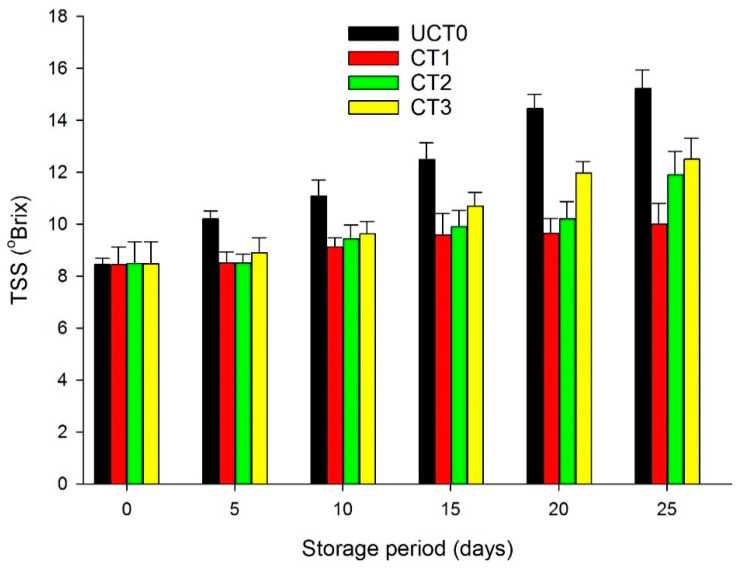
Presence of total soluble solids (°Brix) of Golden Delicious apple slices coated (with CT1, CT2, and CT3), and not treated (UCT0) at day 0, and after 5, 10, 15, 20, and 25-days of storage at 4 °C in dark conditions and 85% relative humidity (RH). Values indicate the means ± standard deviations (*n* = 3). Abbreviations: UCT0, uncoated apple slices used as control; CT1, apple slices coated with chitosan-glycerol-cinnamon oil; CT2, apple slices coated with guar gum-glycerol-cinnamon oil; CT3, apple slices coated with composite guar gum-starch-glycerol-cinnamon oil.

All treatments showed a gradual increase in TSS during storage at 4 °C (Figure 5). TSS at 25-days of storage were higher in UCT0 (*p* < 0.05), while CT1 showed 10%, CT2 showed 12%, and CT3 exhibited 12.5% from the initial TSS which was a mean value of 8.5% for UCT0, CT1, CT2, and CT3. The lowest values for TSS at the end of the storage period were for CT1 followed by CT2 and CT3 showing that these treatments compared with UCT0, provided a semipermeable barrier on the apple slices, modifying the internal atmosphere and changing the CO_2_ production. The ripening in postharvest storage causes variations in TSS as a result of the hydrolytic changes in polysaccharides [12], and can be delayed because of the application of coatings due to the reduction in the metabolic activity of fruits [40,41]. Thakur et al. [6] applied starch coatings to apples and were compared to uncoated samples at 20 °C and 5 °C during a 5-weeks storage trial; the results for 20 °C storage showed a gradual increase in TSS values for uncoated and coated samples and a similar effect was observed at 5 °C, but at a slower rate. Chitosan edible coatings applied on fresh-cut apples stored at 4 °C for 17-days showed a slower increase tendency in terms of TSS values compared to the uncoated samples [42].

### 3.6. Titratable Acidity (TA)

**Figure 6:**
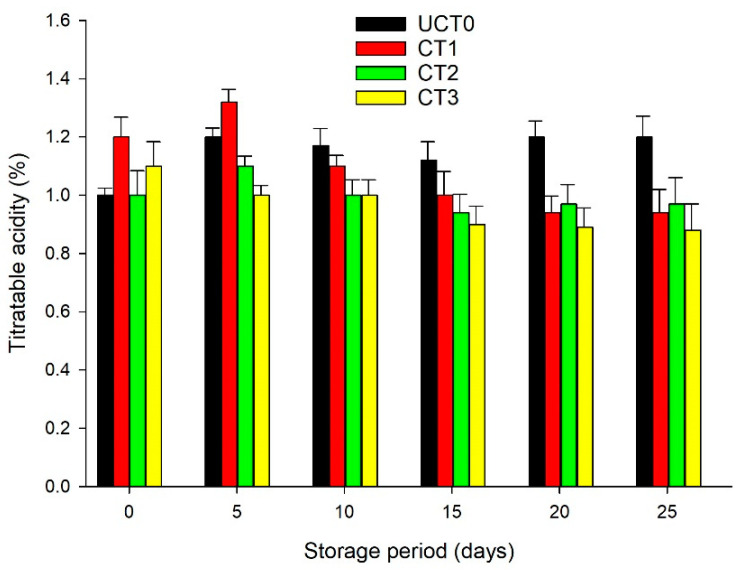
Titratable acidity (%) of Golden Delicious apple slices coated (with CT1, CT2, and CT3), and not treated (UCT0) at day 0, and after 5, 10, 15, 20, and 25-days of storage at 4 °C in dark conditions and 85% relative humidity (RH). Values indicate the means ± standard deviations (*n* = 3). Abbreviations: UCT0, uncoated apple slices used as control; CT1, apple slices coated with chitosan-glycerol-cinnamon oil; CT2, apple slices coated with guar gum-glycerol-cinnamon oil; CT3, apple slices coated with composite guar gum-starch-glycerol-cinnamon oil.

In respiration metabolism, organic acids are used as substrates during the ripening process due to the oxidation reaction of these acids, decreasing TA [8,43]. This effect is exhibited because the malic acid content increases during the ripening process and once the minimally processed apples reach the fully ripe stage, malic acid decreases [44]. The decrease in TA during the storage period reveals the intensification of the respiration rate of the minimally processed apples due to cutting and other processed procedures [8]. CT1, CT2, and CT3 showed lower TA values compared with UCT0, from 10-days of the storage period in cold conditions (Figure 6); similar findings have been reported by Zhelyazkov et al. [40]. They reported that chitosan-acetic-acid-coated apple cubes exhibited slower TA losses than the uncoated apple cubes through the 17-days storage period at 4 °C. CT3 coating proved to be an appropriate method to retard the decrease in TA content from 5-days through the 15-days of the storage period. Romani et al. [40] reported that the decrease in TA values in apple samples coated with a rice starch/protein blend was slower than in uncoated samples as a result of the barrier properties of starch coatings.

Liu et al. [41] reported that chitosan coating with ascorbic acid or calcium chloride helped to preserve the quality content of TA on apple cubes stored at room temperature during a 10-h assay. Cofelice et al. [8] mentioned that TA values of fresh-cut apples tended to decrease during a 14-days assay due to the alginate and lemongrass essential oil coatings applied, extending the shelf-life of the processed fruit.

### 3.7. Microbiology Analysis

Table 1 presents the mesophilic bacteria, yeast, and molds on the apple´s slices subjected to the four treatments. There were differences (*p* < 0.05) between the microbial load of UCT0 and the coated samples (CT1, CT2, and CT3). For mesophilic bacteria, there were differences among treatments during the storage period (Table 1); on 0-day for UCT0 the log CFU/g was 1.60 and on the 25-day, it was 3.25 log CFU/g., as for CT1, the count was 1.58 log CFU/g on 0-day and 2.00 log CFU/g for 25-days, CT2 on 0-day had 1.56 log CFU/g and for 25-days the load was 2.20 log CFU/g. The load for CT3 on 0-day was 1.62 log CFU/g and for 25-days it was 2.10 log CFU/g. When comparing the counts of mesophilic bacteria for the treatment, a difference (*p* < 0.05) was found between CT1, CT2, CT3, and not coating (UCT0) delaying the growth of bacteria. In the case of coated samples, no significant differences were found between treatments. These results provide evidence that the use of any of the three coated treatments delays the growth of mesophilic bacteria on minimally processed apple slices at 4 °C storage for up to 25-days.

For yeast and molds, the log CFU/g differed (*p* < 0.05) among treatments (Table 1) during the storage period. For the UCT0 treatment, the load of log CFU/g was 1.15 on 0-day, while for 25-days, it was 2.17 log CFU/g. For CT1, the yeast and mold load on 0-day was 1.12 log CFU/g and it was 1.74 log CFU/g for 25-days, CT2 presented a load of 1.14 log CFU/g on 0-day and 1.96 log CFU/g for 25-days, and CT3 showed a load of 1.13 log CFU/g on 0-day and 1.85 log CFU/g for 25-days. A difference (*p* < 0.05) was found between UCT0 and the coating treatments, also differences (*p* < 0.05) between coatings were also found. As was expected, a delay in the growth of the yeast and molds was exhibited when comparing UCT0 with CT1, CT2, and CT3. It was also observed that there was a significant difference in the growth retardation response of CT1 compared to CT2 and CT3 (Table 1). So as in the case of mesophilic bacteria, it was evident that the chitosan-based coating was the best to slow down the growth of yeast and molds, followed by CT2, and CT3 (*p* > 0.05).

Zhelyazkov et al. [42] reported the antimicrobial effect of chitosan coatings on apple cubes, sampling every three days for 17-days for mesophilic bacteria and yeast and molds. Carrageenan or whey protein concentrate coatings—both with chemical antibrowning agents—effectively reduced levels of mesophilic bacteria and yeast and molds during a two-week assay [3]. Shellac and aloe vera gel coatings significantly reduced mesophilic bacteria and yeast and molds in apple slices during a storage time of 30-days; however, shellac coatings exhibited a higher antimicrobial effect than the aloe vera coating [45].

### 3.8. Respiration Rate

**Figure 7:**
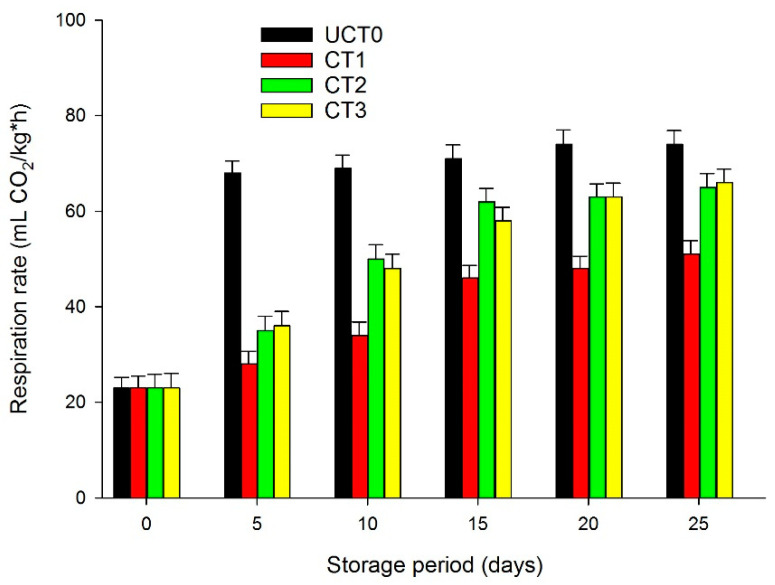
Respiration rate (mL CO_2_/kg*h) of Golden Delicious apple slices coated (with CT1, CT2, and CT3), and not treated (UCT0) at day 0, and after 5, 10, 15, 20, and 25-days of storage at 4 °C in dark conditions and 85% relative humidity (RH). Values indicate the means ± standard deviations (*n* = 3). Abbreviations: UCT0, uncoated apple slices used as control; CT1, apple slices coated with chitosan-glycerol-cinnamon oil; CT2, apple slices coated with guar gum-glycerol-cinnamon oil; CT3, apple slices coated with composite guar gum-starch-glycerol-cinnamon oil.

The respiration process increases when apples are subjected to minimal processes of cutting and peeling due to the physical injury. Thus, it is necessary to reduce the initial respiration rate to extend the shelf-life of apple slices or other minimally processed fruits. The use of edible coatings on the minimally processed apples significantly reduced the respiratory process compared to UCT0 (Figure 7). UCT0 showed the greatest respiration values (*p* < 0.05) as a result of the minimal processes applied and the absence of surface coatings on the tissue of these apple slices. The three coated treatments reduced the respiratory values and delayed the fulfillment of climacteric behavior compared to the uncoated samples. CT2 and CT3 coatings did not differ (*p* > 0.05) regarding the retardation of respiration rate, whereas CT1 showed a significant difference (*p* < 0.05) as an anti-respiratory barrier throughout the 25-days of storage at 4 °C (Figure 7). Thus, CT1 was more effective to extend the shelf-life of Golden Delicious apple slices.

Qi et al. [11] reported that chitosan with antibrowning chemical agents decreased the respiratory rate of apple slices at 5 °C during an 8-days trial. Chauhan et al. [45] stated that shellac or aloe gel coating significantly suppressed the respiration rate of apple slices during a 30-days assay at 6 °C, so our findings are in agreement with these authors in the sense that the use of coatings decreased the respiration rate in minimally processed apple slices.

### 3.9. Sensory Analysis

**Figure 8:**
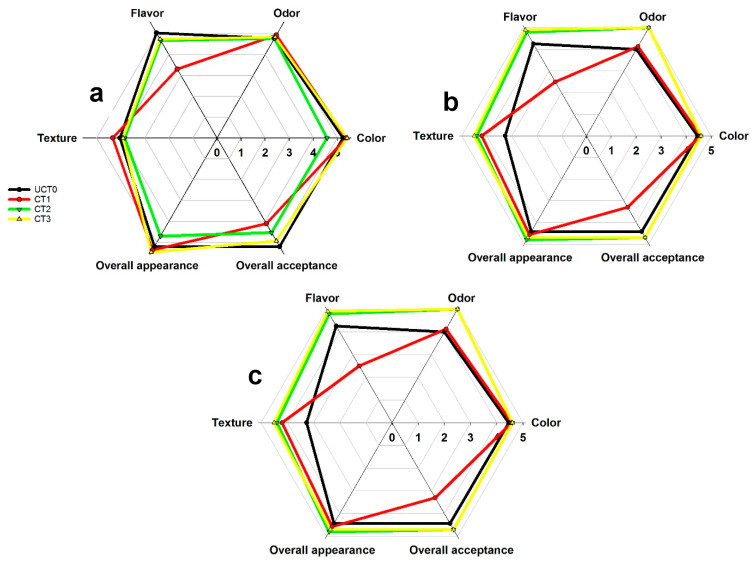
Sensory analyses of Golden Delicious apple slices coated (with CT1, CT2, and CT3), and uncoated at 0 (**a**), 5 (**b**), and 10 (**c**) days of storage at 4 °C in dark conditions and 85% relative humidity (RH). Values indicate the means for the following attributes: color, odor, flavor, texture, global appearance, and global acceptance. Abbreviations: UCT0, uncoated apple slices used as control; CT1, apple slices coated with chitosan-glycerol-cinnamon oil; CT2, apple slices coated with guar gum-glycerol-cinnamon oil; CT3, apple slices coated with composite guar gum-starch-glycerol-cinnamon oil.

Sensory evaluation is primarily a source of product information, such as the distinguishing attributes that are important quality variables to assure the acceptability of it by consumers. In Figure 8a–c, the highest scores for the sensory evaluation were obtained with the coated treatments as compared to UCT0. The evaluation was done only on the initial day of the trail and on 5-days and 10-days due reasons for the safety of the panelists, based on ISO 4833:2003E [46], RM615-2003 [47] (mesophilic bacteria), and ISO 21527-1:2003E [48] (yeast and molds). Figure 8a corresponds to the evaluation of the initial day. There was a significant difference (*p* < 0.05) in flavor and overall acceptance between UCT0 and CT1 due to the presence of chitosan in the formulation of CT1, which caused that some of the panelists referred to it as a not acceptable flavor for these samples, and maybe the difference on overall acceptance attributed to the flavor and the visual aspects. In Figure 8b (5-days) and Figure 8c (10-days) significant differences (*p* < 0.05) were more relevant, particularly in UCT0 and CT1, because as time passed several attributes were affected. For CT2 and CT3, no significant difference was detected (*p* > 0.05) for the sensory attributes tested. Considering the sensory results of the present study, CT2 and CT3 are recommended to extent the shelf-life of minimally processed apples; CT1 is not recommended because of the slightly unpleasant flavor of chitosan. Several authors that have used chitosan coatings applied on different food matrices have not mentioned any results referring to unpleasant flavor or odors [11,16,17,40].

Lee et al. [3] showed that the edible coatings of carrageenan or whey protein concentrates received higher scores than uncoated apples, and the main difference among these can be attributed to the off-flavor of the samples. Supapvanich et al. [49] reported that aloe gel coatings retarded the BI and the overall acceptability of the sliced apple, in contrast with the uncoated samples.

## 4. Conclusions

The edible coatings of chitosan-glycerol-cinnamon essential oil (CT1), guar gum-glycerol-cinnamon essential oil (CT2), and composite guar gum-starch-glycerol-cinnamon essential oil (CT3) delayed the loss of weight, firmness, phenolic content, acidity, and sensory attributes, and at the same time retarded the increase of the brown index, respiration rate, and microbial growth throughout the study (25-days), when compared with uncoated apple slices. Consequently, the use of edible coatings with cinnamon essential oil (CT1, CT2, and CT3) has proven to be a promising alternative. The bioactive coating of chitosan-glycerol-cinnamon essential oil proved to be most effective in several quality variables and shelf-life extension, although it obtained a slightly unpleasant acceptability in terms of the flavor attribute.

## Figures and Tables

**Table 1:** Effect of coating on the mesophilic bacteria, yeast, and mold counts in the minimally processed Golden Delicious apple slices stored at 4 °C for 25-days.

Microorganisms	Storage Period (days)	Treatments			
		UCT0	CT1	CT2	CT3
Mesophilic bacteria (log CFU/g)	0	1.60 ^d A^	1.58 ^d A^	1.56 ^d A^	1.62 ^d A^
	5	2.19 ^c A^	1.72 ^c B^	1.81 ^c B^	1.79 ^c B^
	10	2.32 ^b c A^	1.83 ^b c B^	1.95 ^b c B^	2.02 ^b c B^
	15	2.49 ^b c A^	1.88 ^b c B^	2.01 ^b c B^	2.00 ^b c B^
	20	2.62 ^a b A^	1.92 ^a b B^	2.10 ^a b B^	2.09 ^a B^
	25	3.25 ^a A^	2.00 ^a B^	2.20 ^a B^	2.10 ^a B^
Yeast and molds (log CFU/g)	0	1.15 ^d A^	1.12 ^d A^	1.14 ^d A^	1.13 ^d A^
	5	1.77 ^c A^	1.46 ^c C^	1.57 ^c B^	1.54 ^c B^
	10	1.90 ^b A^	1.58 ^b C^	1.71 ^b c B^	1.77 ^b B^
	15	2.10 ^a b A^	1.62 ^b C^	1.77 ^b c B^	1.75 ^b B^
	20	2.20 ^a A^	1.66 ^a b C^	1.86 ^b B^	1.84 ^a B^
	25	2.17 ^a A^	1.74 ^a C^	1.96 ^a B^	1.85 ^a B^

UCT0, uncoated apple slices used as control; CT1, apple slices coated with chitosan-glycerol-cinnamon oil; CT2, apple slices coated with guar gum-glycerol-cinnamon oil; CT3, apple slices coated with composite guar gum-starch-glycerol-cinnamon oil. For treatments, means with different uppercase letters in the same row differ (*p* < 0.05). For the storage period, means with different lowercase letters in the same column differ (*p* < 0.05).
